# Research Progress of Low-Voltage Areas Associated with Atrial Fibrillation

**DOI:** 10.31083/j.rcm2411320

**Published:** 2023-11-17

**Authors:** Yunfei Gu, Yang Shao, Songsen Li, Tong Liu

**Affiliations:** ^1^Department of Cardiology, Luoyang Central Hospital Affiliated to Zhengzhou University, 471000 Luoyang, Henan, China; ^2^Department of Cardiology, Tianjin Institute of Cardiology, Second Hospital of Tianjin Medical University, 300091 Tianjin, China; ^3^The Department of Cardiology, Sahlgrenska University Hospital, 41345 Gothenburg, Sweden

**Keywords:** atrial fibrillation, low-voltage areas, catheter ablation, atrial fibrosis

## Abstract

Atrial fibrosis is an independent predictor of the recurrence of atrial fibrillation (AF) after catheter ablation. Low-voltage areas (LVA) measured during catheter ablation for AF are a commonly used surrogate for the presence of atrial fibrosis. LVA are associated with clinical outcomes and comorbidities and have links to triggering sites for AF. Several trials have shown promising data of targeting ablation in LVA, however the results have been mixed. This article will review the role of LVA in the prediction of adverse events in AF patients, including stroke, how to predict the presence of LVA, and the impact of LVA ablation on the recurrence of AF.

## 1. Introduction

Atrial fibrillation (AF) is a common cardiac arrhythmia that is closely associated with adverse events such as ischemic stroke, heart failure, death, and decreased quality of life [1]. Heart rate control and rhythm control are two strategies for the treatment of AF. Rhythm control has been shown to reduce the recurrence of atrial arrhythmias and composite cardiovascular endpoint events compared to rate control [2]. Clinical studies and meta-analyses have shown that catheter ablation is superior to antiarrhythmic drugs in maintaining sinus rhythm, avoiding side effects of bradycardia and arrhythmias caused by antiarrhythmic drugs, and improving clinical outcomes [3, 4, 5]. However, the recurrence of AF after ablation remains high. Many patients require several procedures of catheter ablation [6, 7, 8] with the development of new ways of catheter ablation being needed.

The presence of atrial fibrosis is a predictor of AF recurrence after radiofrequency ablation [9]. Such atrial fibrotic areas usually have low voltage by intracardial voltage mapping. A low-voltage area (LVA) is defined as an area with bipolar voltage <0.5 mV by intracardial voltage mapping. Ablation in those LVAs has been shown to modify the atrial substrate and improve the success rate of AF ablation. This article will review the role of LVA in the prediction of adverse events in AF patients, including stroke, how to predict the presence of LVA, and the impact of LVA ablation on AF recurrence.

## 2. The Role of LVA in the Prediction of Adverse Events in AF

AF has a five-fold increased risk of thromboembolic events. A silent cerebral ischemia can be found in up to 90% of AF patients on cerebral delayed-enhancement magnetic resonance imaging (MRI). A powerful risk stratification is therefore crucial for patients with AF. The current ${\mathrm{CHA}}_{2}$${\mathrm{DS}}_{2}$-VASc risk stratification is shown to have only moderate ability to evaluate the risk of stroke in patients with AF. Atrial remodeling is a genesis of AF recurrence and progression. LVA can be used to estimate atrial remodeling by the degree of atrial fibrosis. A wide area of LVA indicates a broad range of atrial fibrosis, which is associated with higher rates of thromboembolic events and AF recurrence. In a retrospective study of 200 patients with AF [10], patients with stroke or asymptomatic cerebral infarction on MRI findings had a larger area of ​​atrial LVA. LVA was shown to be independently associated with the presence of thromboembolic events. The inclusion of LVA in ${\mathrm{CHA}}_{2}$${\mathrm{DS}}_{2}$-VASc risk stratification might increase the predictive power for thromboembolic events. This could benefit individuals with lower ${\mathrm{CHA}}_{2}$${\mathrm{DS}}_{2}$-VASc. A recent single-center study selected patients with ${\mathrm{CHA}}_{2}$${\mathrm{DS}}_{2}$-VASc score of 0–1 and demonstrated that patients with a history of embolism had a higher proportion of left atrial LVAs [11]. In this study, LVA were mostly found in the anterior wall and anterior LVA was an independent predictor of thromboembolic events. In patients with low ${\mathrm{CHA}}_{2}$${\mathrm{DS}}_{2}$-VASc score but with LVA present, anticoagulation treatment may be considered in clinical practice. Although it is unclear why LVA can be an independent predictor of embolic events, it has been shown that LVA on the anterior atria wall is associated with low blood flow in the atrial appendage. In addition, when LVA locates near the atrial orifice, it is related to further lower blood flow in the atrial appendage. The reduction in the left atrial appendage blood flow velocity is recognized as genesis for the development of emboli, increasing the risk of stroke. It appears that LVA increases the incidence of thrombotic events by decreasing the blood flow velocity of the left atrial appendage [12].

In addition, the presence of LVA, especially >10%, has been shown to predict AF recurrence after radiofrequency catheter ablation in patients with AF [9, 13]. Recurrence rate of AF in patients with LVA within one year has been shown to be significantly higher than that of patients without LVA [14].

Moreover, extensive LVA seems to be associated with sinus node dysfunction. A large retrospective study that included more than 1200 patients who underwent catheter ablation for persistent AF found that 3.2% of those patients needed pacemaker implantation because of sinus node dysfunction [15]. LVA are identified in the same study as strong predictors for acute cardiac pacing.

## 3. How to Predict the Existence of LVA

Presently, intracardiac voltage mapping has emerged as a reliable modality to detect abnormal atrial substrates. However, the standardization of voltage mapping in the detection of LVA is still lacking. The number of voltage points during mapping can vary from 90 to 2566 points per left atrium [16], leading to different mapping resolution and therefore variable capacity in the detection of LVA. Most studies utilize high-density bipolar mapping catheters, which generally collect larger volume of mapping points than the catheters used in earlier studies. However, it is essential to verify those collected points by excluding incorrectly annotated signals in the presence of atrial ectopy, uncaptured pacing, noise, and ventricular and atrial far-field sensing. Radoslaw M *et al*. [17] excluded the tubular and antral portions of pulmonary veins (PVs) inside the ablation encirclement and left atrial appendage from the total atrial area, which could be one of the reasons explaining their different LVA percentage from the other studies. Moreover, different electrodes and catheters used in high-density voltage mapping can generate various degrees of LVA. Masaharu *et al*. [18] have compared a direction-independent grid catheter with a conventional circular mapping catheter in the measurement of LVA area. They found that the grid catheter took less time in collecting mapping points and had better tissue contact during voltage contact, which leads to higher voltages collected during mapping and therefore less area of LVA compared to the circular catheter. This could explain why the LVA identified in specific studies could be reclassified as normal tissue in other studies. Furthermore, the voltage cut-off during mapping is a key to defining how much LVA is detected. Diverse cut-off values, e.g., 0.1–1.5 mV used in previous studies, lead to different calculated LVA area percentages [13, 18, 19]. However, no cut-off value has been validated due to the lack of histological examination for verification of fibrosis. At present, a cut-off value <0.5 mV is commonly used for fibrotic tissue and <0.1 mV for dense scarring [16].

Rhythm during voltage mapping is another factor that affects LVA detection. Voltage mapping can be done before cardioversion of AF (AF rhythm) or after cardioversion of AF (sinus rhythm). Ndrepepa *et al*. [20] found that voltages in the atria were lower during AF than during sinus rhythm. Coronary sinus pacing may normalize the low voltage levels in LVA. The cut-off value <0.2 mV has been proposed for the identification of LVA during AF corresponding to the cut-off value <0.5 mV during sinus rhythm. However, tachycardia itself is not wholly responsible for lowering atrial voltage but that continually changing electrical activation direction during AF. In patients with both AF and atrial flutter, voltage mapping may record higher voltages in the LVA (less LVA) at atrial flutter than during sinus rhythm. Also, duration of AF has shown no correlation with the detection of LVA [17, 21]. Clearly, persistent AF is not a marker of LVA.

Voltage mapping before and after pulmonary vein isolation (PVI) is another way to affect LVA detection. Post-PVI can reduce the overall atrial scar burden and therefore voltage mapping may miss the atrial LVA in the pulmonary vein antrum, giving lower detection of LVA and therefore a smaller extent of LVA [17, 18]. Moreover, factors that affect the collection of bipolar electrograms can influence the measurement of LVA extent. Bipolar electrograms demonstrate voltage difference recorded by two closed placed electrodes. Therefore, the affecting variables can be interelectrode spacing, tissue contact, filtering, mapping density and resolution, and activation vector.

Non-invasive methods have been studied in the detection of LVA. A systemic review of MRI has shown a correlation between late gadolinium enhancement (LGE) in MRI and voltage mapping in the detection of LVA [16]. Measurement of the plasma level of suppression of tumorigenicity 2 (ST2) could be a potential way to predict LVA. Patients with LVA area >20% have been shown to have higher plasma soluble ST2 which has been shown to be an independent predictor of atrial LVA presence [22]. Similarly, inflammatory factors have been shown to be a predictor of LVA existence, probably because inflammation is involved in the development of LVA [23]. Patients with LVA had a larger proportion of intermediate monocytes and higher expression levels of Toll-like receptor-4. Regression analysis has shown that the proportion of intermediate monocytes is an independent predictor of the presence of atrial LVA. In addition, B-type natriuretic peptide (BNP) level [24] and clinical features [17, 25, 26] (elderly, female, increased left atrial surface area, persistent AF, dilated cardiomyopathy, hypertrophic cardiomyopathy, and sinus node dysfunction) have been shown to be associated with the development of atrial LVA.

As a simple, fast and economical examination method, electrocardiogram (ECG) is also valuable in predicting LVA. The amplitude of P wave in lead I has been shown to negatively correlate with the presence of LVA, while the duration of P wave in some leads may be positively correlated with the presence of LVA [27]. When 0.062 mV was used as a cut-off, its sensitivity and specificity in predicting LVA >35% (severe fibrosis in the UTAH classification [Utch university’s classification method for atrial fibrosis]) were reported to be 85% and 88%, respectively. However, some researchers found that the predicting power of P-wave amplitude for LVA is weak [28]. Ooie T *et al*. [29] found that the LVA positive group mostly had P-wave duration ≥120 ms with biphasic P waves in inferior leads, whereas the LVA negative group had shorter P-wave duration <120 ms. A combination of P waves of ≥120 ms and biphasic P waves in any inferior lead achieves predictive power of 83% sensitivity and 98% specificity for the presence of LVA. P-wave duration and biphasic morphologies have been shown to be independent predictors of LVA. In addition, the PR interval (A parameter of electrocardiogram, the time from the start of p-wave to the start of QRS wave) under sinus rhythm can also predict the existence of LVA [30].

Several scoring systems have been developed to predict LVA, such as the DR-FLASH score (diabetes, renal dysfunction, persistent AF, left atrial diameter >45 mm, age >65 years, female and hypertension) [31], SPEED score (including gender, persistent AF, age >70 years, increased brain natriuretic peptide and diabetes) [32] and the improved APPLE score (age, type of AF, estimated glomerular filtration rate (eGFR), left atrial volume and left atrial ejection score) [33]. However, how to widely use these scoring systems clinically requires more research and trials.

## 4. Effect of Ablation in LVA on AF Recurrence

Rolf *et al*. [34] were the first group to perform LVA ablation in patients with AF. They demonstrated that PVI combined with LVA ablation reduced the recurrence of AF/atrial tachycardia. The study by Jadidi *et al*. [35] revealed that PVI together with LVA ablation had a higher successful rate of ablation, defined as no arrhythmia within one year, 69% vs 47%, *p* <0.001. Similarly, Kottkamp achieved 83.3% of no AF recurrence with one year with PVI and Box isolation of LVA [35, 36]. A meta-analysis that included [37] 800 patients with AF (92% were non paroxysmal AF) showed similar results. After an average follow-up of 17 months, the proportion of no AF/atrial tachycardia in PVI combined with LVA ablation was significantly higher than that in PVI alone (70% vs 43%, OR = 3.41). The operation time, fluoroscopy time and ablation time were shorter in the PVI+LVA ablation group, while the incidence of adverse events was not increased in the PVI+LVA group.

In the study of Kircher *et al*. [38], PVI plus LVA ablation were administered to patients with AF regardless of whether the AF was paroxysmal or persistent. After 1-year follow-up, the proportion of no arrhythmia in the PVI+LVA ablation group was found to be significantly higher than that in the control group (68% vs 42%, *p* = 0.003). The suggestion is that individualized ablation under the guidance of LVA may be superior to conventional ablation strategy. It can be done by adding LVA ablation if traditional PVI is not able to terminate AF. This would result in 40% of patients with AF converting to sinus rhythm with a decreased recurrence [39]. This suggests that additional LVA ablation can increase the success rate of AF ablation. However, the STABLE-SR study and its follow-up STABLE-SR-II study have shown diverse results [40, 41]. The investigators were not able to reduce the recurrence of arrhythmia by adding LVA ablation to the traditional PVI. The different result could be due to the severity of fibrosis or the extent of LVA. The prevalence of LVA has been shown to be 10–63% in paroxysmal AF and 35–100% in persistent AF [42]. Takanori *et al*. [43] have classified LVA into four stages based on extension of LVA. PVI plus LVA ablation did not reduce AF recurrence or improve clinical outcomes in patients with extensive LVA (LVAs >30% of total left atrial surface area). AF substrate has been suspected as not always localizing within LVAs. Instead, LVA is supposed to be a surrogate of global structural remodeling and not a local phenomenon. Takanori *et al*. [19] have shown that voltage reduction in the LA is a diffuse process associated with fibrosis. Presence of LVAs reflects diffuse voltage reduction of the LA, suggesting a diffuse fibrotic remodeling rather than local changes in the atria. In this sense, extra ablation locally in LVAs beyond PVI would not lead to reduced AF recurrence [44]. However, a recent meta-analysis [45] and a randomized trial seem to bring back new hope for ablation in LVA. The meta-analysis demonstrated that PVI plus LVA ablation in persistent AF reduced the recurrence of AF without increasing the overall operation time and the incidence of complications. Similarly, in the randomized trial, PVI plus individualized ablation of LVA significantly improved outcomes in patients with persistent AF [46].

## 5. Conclusions

Atrial remodeling is a genesis of AF recurrence after ablation. LVAs in the atria, a surrogate of atrial remodeling, are highly associated with thromboembolic events. This can be a potential factor added to the traditional ${\mathrm{CHA}}_{2}$${\mathrm{DS}}_{2}$-VASc score to increase the predictive power for thromboembolic events. Moreover, catheter ablation in LVA may be a potential method that deserves to be developed in addition to traditional pulmonary vein isolation, in order to increase the success rate of ablation and reduce the recurrence of AF.
